# Overreliance on Radiological Findings Leading to Misdiagnosed Giant Retroperitoneal Ganglioneuroma: A Case Report and Literature Review

**DOI:** 10.7759/cureus.43914

**Published:** 2023-08-22

**Authors:** Dharmendra Shah, Shivani R Chaudhary, Shahin Khan, Shashwat Mallik

**Affiliations:** 1 General Surgery, Medical College Baroda, Vadodara, IND

**Keywords:** exploratory laparotomy, mesenteric cyst, abdominal tumor, pediatric surgery, retroperitoneal, ganglioneuroma

## Abstract

Ganglioneuroma is a rare, benign, well-differentiated neurogenic tumor most commonly located in the posterior mediastinum or retroperitoneum. Giant ganglioneuromas are even less common; this is only the 19th reported case in literature to date. We present a case of a giant retroperitoneal ganglioneuroma in a five-year-old child, which on imaging mimicked a mesenteric cyst and posed various challenges in its management. Histopathology later confirmed our misdiagnosis and revealed the tumor to be a ganglioneuroma. This unique case serves as a lesson for clinicians to not operate before receiving histopathological confirmation of their diagnosis.

## Introduction

Ganglioneuroma is a rare neurogenic tumor with a prevalence of one per million population. Usually, ganglioneuromas are very slow-growing, and they hardly ever cause symptoms. Symptomatic cases are those where the mass effect of the growth is seen [1,2]. We present a case of a symptomatic giant ganglioneuroma which was initially misdiagnosed due to over-reliance on radiological investigations. This is the first case that sheds light on the importance of histopathological confirmation prior to surgical intervention in patients with giant ganglioneuromas.

## Case presentation

**Figure 1:**
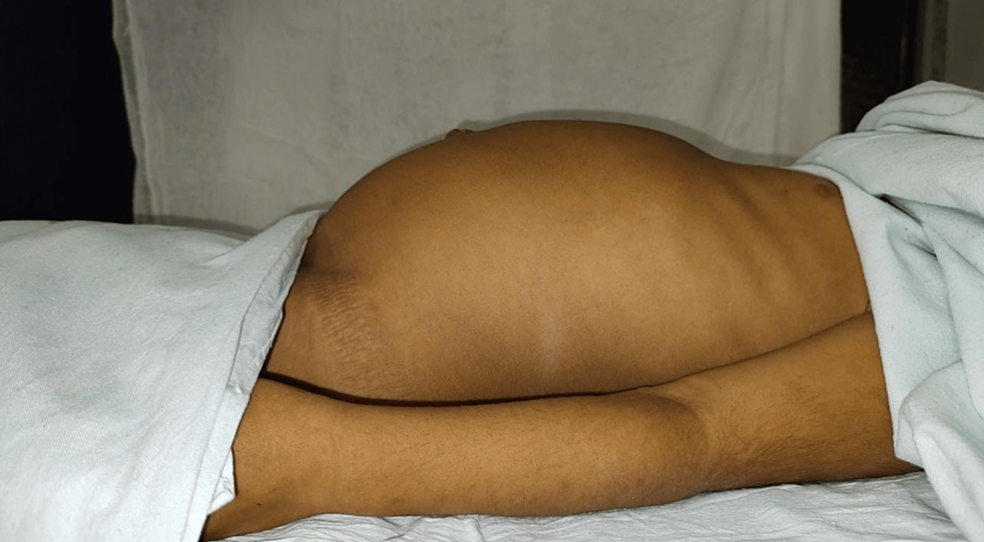
Lateral view of the distended abdomen

**Figure 2:**
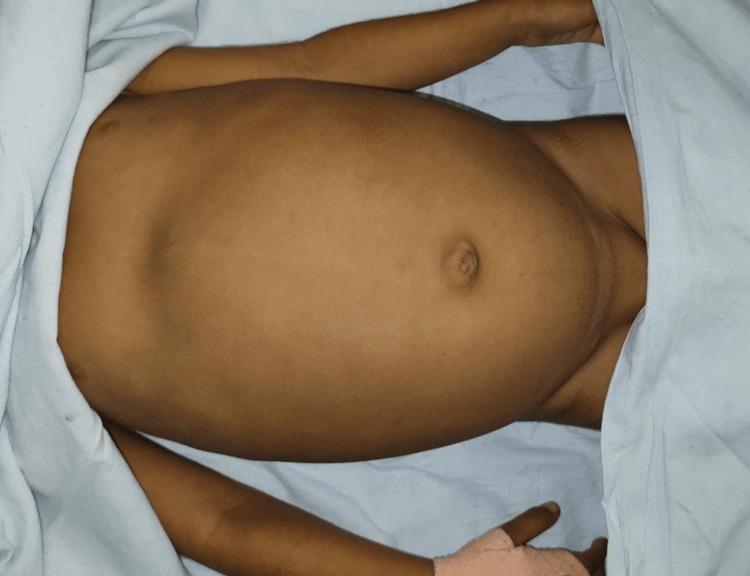
Anterior view of the distended abdomen

A five-year-old girl was brought by her parents with complaints of abdominal distension and generalized abdominal pain for five months. On examination, the patient was vitally stable, fairly built, and well-nourished, with no family history of any abdominal masses or neoplasms. Abdominal examination revealed a single, 12×10 cm sized oval lump, freely movable in both horizontal and vertical axes, firm in consistency and dull on percussion in the epigastric, umbilical, hypogastric, left lumbar, and left iliac fossa quadrant (Figures 1, 2). Contrast-enhanced computed tomography (CECT) of the abdomen revealed a large lobulated heterogeneously enhancing mass of 7.7 (anteroposterior)×10.4 (transverse)×11.6 (craniocaudal) cm size of soft tissue density, from epigastrium to the pelvis.

**Figure 3:**
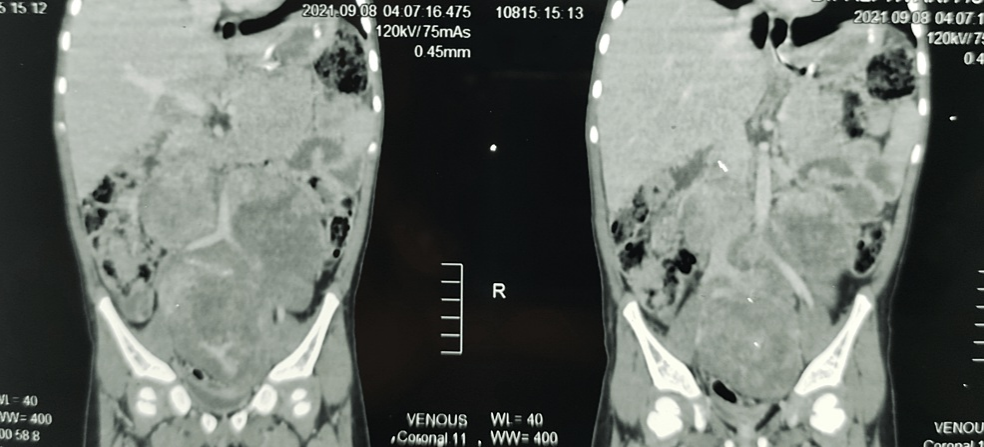
CECT abdomen (coronal section) CECT: Contrast-enhanced computed tomography

**Figure 4:**
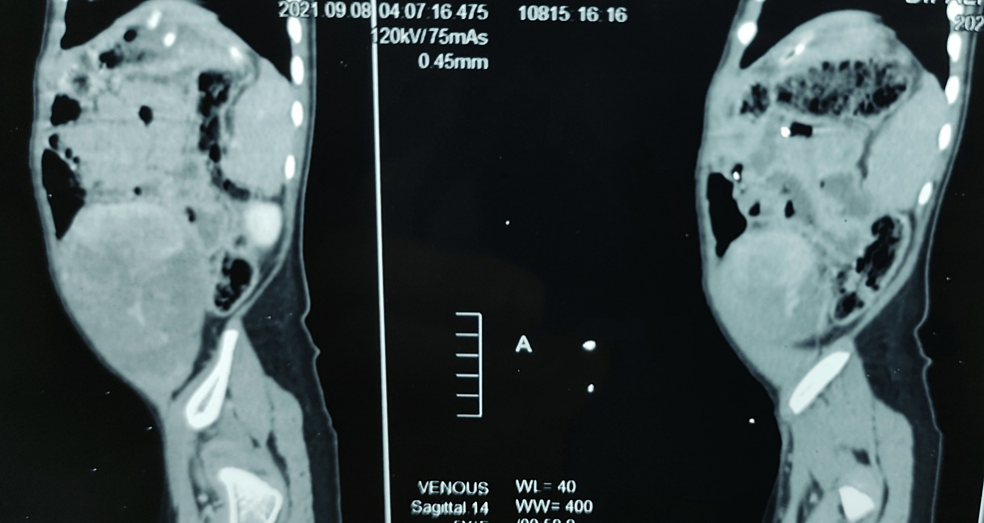
CECT abdomen (sagittal section) CECT: Contrast-enhanced computed tomography

It was 360-degree encasing and splaying the bilateral common iliac vessels and compressing the inferior vena cava (Figures 3, 4). However, it was not invading any structures and was suggestive of a benign neoplasm of mesenchymal origin. As on clinical examination, the tumor was found to be firm in consistency and was freely mobile in both axes and the CT findings commented that it was most likely a mesenteric cyst; subtotal resection of the tumor was planned by a team of pediatric surgeons and general surgeons. The part of the tumor encasing major vessels was planned to be left behind, as subtotal resection was deemed to be appropriate management for this condition.

**Figure 5:**
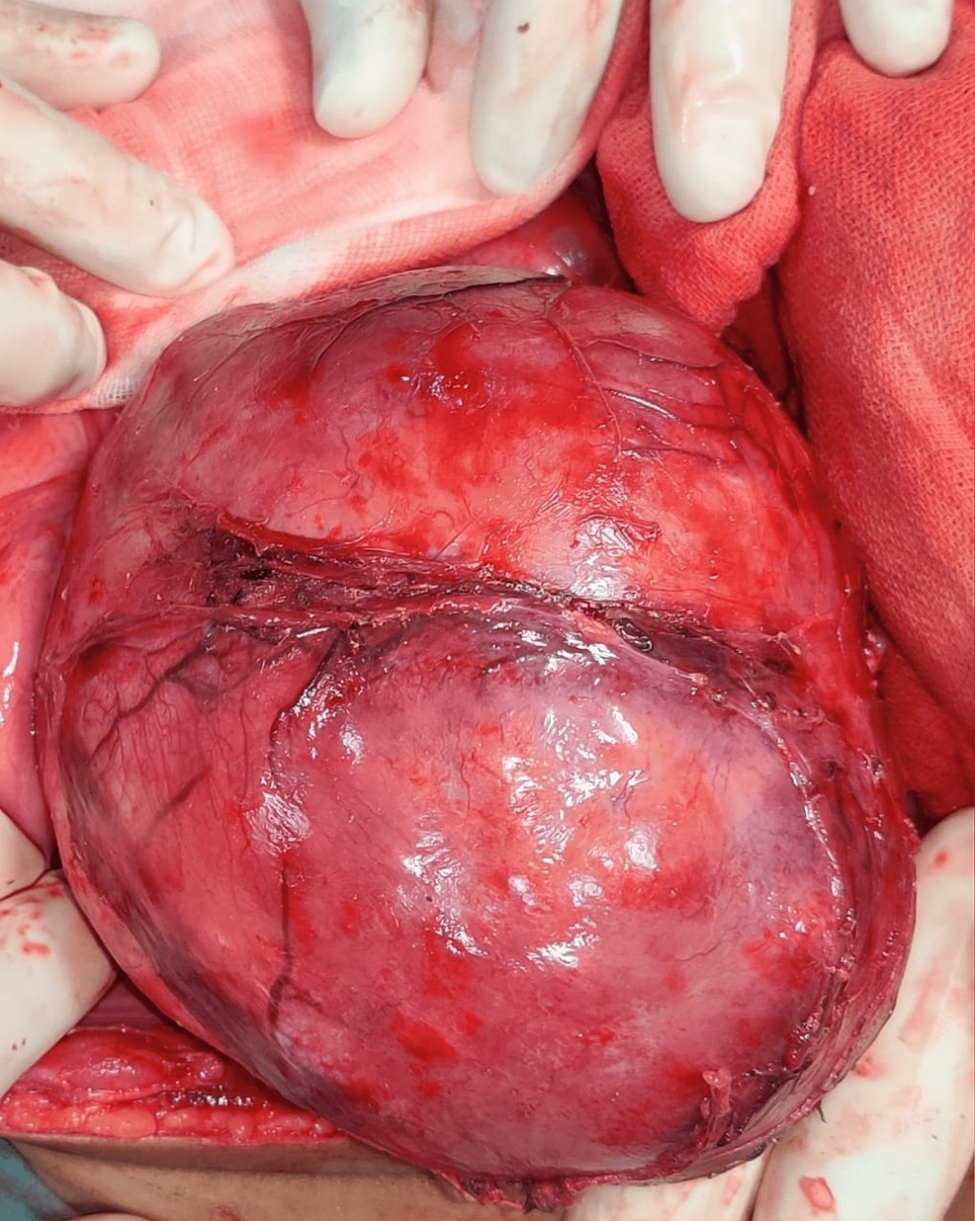
Tumor as seen during surgical exploration

**Figure 6:**
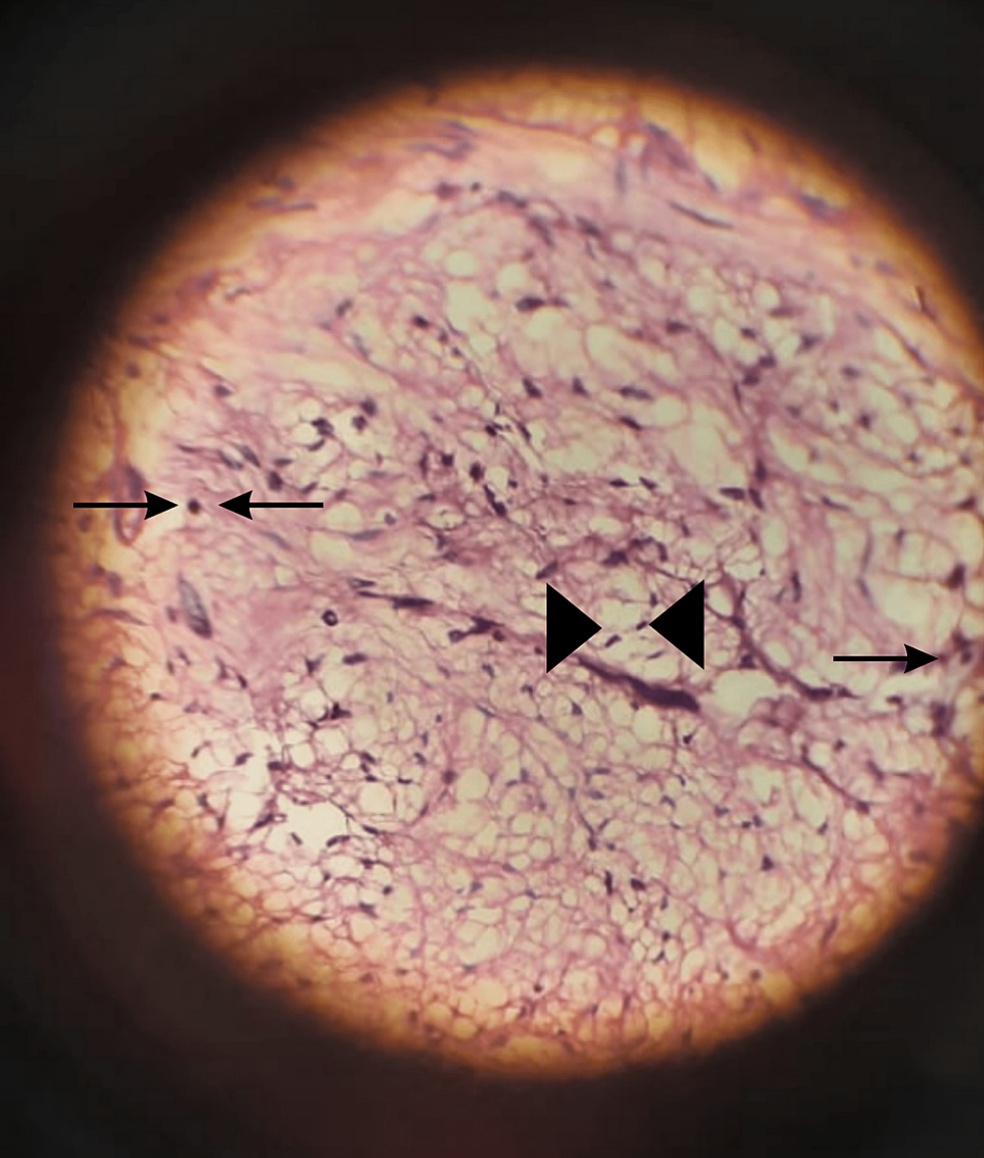
Histopathological slide of the tumor Arrows - Ganglion cells; Arrow Heads - Schwann cells (spindle-shaped)

Surgical exploration under general anesthesia was done by a team comprising a general surgeon, a pediatric surgeon, and a vascular surgeon. A massive tumor of approximately 15×10×10 cm was found extending from the epigastrium to the pelvis (Figure 5). As the tumor was adherent to the inferior vena cava and abdominal aorta, and encased the bilateral common iliac vessels 360 degrees with splaying of vessels, it appeared to be a non-resectable retroperitoneal teratoma, a malignant neoplasm. A biopsy of 2×2×2 cm was taken. A histopathological examination later revealed the tumor to be a ganglioneuroma with distinct ganglion cells and Schwannian stroma (Figure 6). The postoperative period was uneventful. The patient was referred to a higher center with a team of surgical oncologists for a second opinion on subtotal or near-total resection, but she was lost to follow-up.

## Discussion

Ganglioneuromas are extremely rare tumors and are usually seen in the posterior mediastinum, retroperitoneum, and adrenal glands [3]. Primary ganglioneuromas are usually seen in late childhood while secondary ganglioneuromas arising from neuroblastomas are more commonly present in young pediatric patients. Ganglioneuromas are mostly benign in nature and their size gradually increases over a rather long period of time with the mean age of presentation being seven years; thus, they are called slow-growing tumors [4]. While most ganglioneuromas are incidentally detected, some present with pressure symptoms like abdominal pain and distension, back pain, intestinal obstruction with vomiting, paraesthesias, and abnormal gait due to a giant ganglioneuroma (any one dimension >10 cm) impinging on surrounding structures [5-7]. This case reports only the 19th giant ganglioneuroma recorded in the literature, as observed by a literature review using PubMed Central (Table 1).

Ganglioneuromas constitute only 0.72%-1.6% of all primary retroperitoneal tumors [1,3]. Most retroperitoneal ganglioneuromas have been found incidentally during regular health checkups or during the investigation of different diseases [5]. Tumor mass is rarely palpable on physical examination, which means ganglioneuromas are usually missed on routine physical check-ups [6]. Radiological investigations such as CECT and MRI should be done to know the size, precise location, shape, presence of calcification, and relation to adjacent structures. On radiological investigation, most retroperitoneal ganglioneuromas have been found to be non-invasive, oval, and well-circumscribed with or without lobules. In a few cases, punctate calcifications have also been reported [5,6]. Preoperative radiology may aid in differentially diagnosing the benign behavior of ganglioneuromas and thus, establish the roadmap for the treatment plan ahead. In addition, it is indispensable to determine the proximity and intimate relationship of the tumor with the major neurovascular structures and viscera. However, radiological imaging could be confusing; hence, histopathology remains the gold standard for its diagnosis [3,5]. On histopathological examination, intersecting bundles of spindle cells are seen with the proliferation of well-differentiated, mature ganglion cells and Schwannian stroma. Immunohistochemistry shows positive staining with S-100 and Neuron Specific Enolase [3]. 

The prognosis of ganglioneuroma is excellent and surgical excision of the tumor is the only required treatment modality. Laparoscopic and robotic surgeries are preferred over open surgeries for small tumors without vessel invasion [3,4]. Subtotal resection should be preferred for ganglioneuromas encasing the vessels and surrounding structures, as the survival rates between complete or incomplete excision are comparable [8]. Both total and partial resections have been successfully performed even for giant ganglioneuromas (Table 1). However, as evident from this case, the decision to operate has to be made by correlating all the clinical, radiological, and histopathological findings instead of over-reliance on radiological findings, which may not be conclusive or even misleading. In such cases, preoperative CT-guided biopsy would avoid unnecessary surgery. Patients would be better managed in facilities equipped with a cath lab and a team consisting of a pediatric oncological surgeon, vascular surgeon, pediatric anesthetist, and pediatrician. Monitoring and regular follow-up are recommended for cases of subtotal excision if the size of the remaining tumor is >2 cm [4,6]. While there is a belief that adjuvant radiotherapy can lead to the malignant transformation of an otherwise benign ganglioneuroma, there is no literature to support this hypothesis. This should prompt an investigation into using adjuvant radiotherapy in case of high-risk surgical excision.

**Table 1:** Giant ganglioneuromas reported in the literature to date TR: Total resection; PR: Partial resection

Sr. No.	Author	Year	Age (years)	Sex	Dimensions (measured by suture material)	Preoperative Biopsy	Procedure	Surgical Complications	Location
1.	Nishinari et al. [9]	2003	33	Male	33×21×12 cm	Not done	Open TR	No	Brazil
2.	Singh et al. [3]	2006	37	Female	11×9×4.5 cm	Not done	Open TR	No	India
3.	Komai et al. [10]	2006	35	Male	22×20×8 cm	Not done	Open TR	No	Japan
4.	Lamichhane and Dhakal [11]	2006	18	Male	15×10cm	Not done	Open PR	No	Nepal
5.	Gándara et al. [12]	2010	36	Female	15 cm	Not done	Open TR	No	Spain
6.	Lai et al. [13]	2011	53	Male	19 cm	Not done	Open TR	No	Taiwan
7.	Lynch et al. [14]	2013	42	Female	12×6×8 cm	Not done	Open TR	No	Ireland
8.	Esen et al. [15]	2015	11	Female	11×6×9 cm	Not done	Open TR	No	Turkey
9.	Kumar et al. [16]	2016	15	Female	11×5.5×6 cm	Not done	Open TR	No	India
10.	Yang et al. [17]	2016	12	Female	13×8×6 cm	Not done	Open PR	No	China
11.	Proposito et al. [18]	2016	42	Female	14.5×11.6×6.5 cm	Not done	Open TR	No	Italy
12.	Rahnemai‑Azar et al. [19]	2017	21	Male	21.5×18.1×7.8 cm	Done; revealed ganglioneuroma	Open TR	No	United States of America
13.	Zheng et al. [20]	2019	4	Male	10.7×17.3×15.5 cm	Not done	Open PR	No	China
14.	Kirchweger et al. [7]	2020	30	Female	35×25×25 cm	Done; revealed ganglioneuroma	Open TR	No	Austria
15.	Falcone et al. [21]	2021	15	Male	15×7.5 cm	Not done	Open TR	No	Italy
16.	AlShammari et al. [22]	2021	29	Female	6.318×22 cm	Done; revealed ganglioneuroma	Open PR	No	Saudi Arabia
17.	Kitazawa et al. [23]	2021	40	Female	11×7 cm	Not done	Open TR	No	Japan
18.	Kora et al. [24]	2021	14	Female	17.3×6.7×5.9 cm	Done; revealed ganglioneuroma	Open TR	No	Morocco
19.	Current study	2022	5	Female	15×10×10 cm	Not done	Open exploratory	No	India

Taking into consideration the previous 18 giant ganglioneuromas reported in the literature, it is observed that only eight are larger (in at least one dimension) than the one seen in our patient. The largest was reported by Kirchweger et al. and measured a massive 35×25×25 cm [7]. Moreover, it is clearly evident that our patient is the second youngest to develop a giant ganglioneuroma, with the youngest age of presentation being four years and the oldest being 53 years. There is a slight female preponderance with only seven male patients out of the total of 19 patients. Four patients underwent partial resection of the tumor while the others had their ganglioneuroma completely removed. Finally, in 15 out of the 19 cases, a preoperative biopsy to confirm the diagnosis of ganglioneuroma was not done. This underlines the fact that it is not common practice to histopathologically confirm the diagnosis before operating on a ganglioneuroma (Table 1). However, the current case emphasizes the importance of a preoperative biopsy to avoid the dangers associated with a misdiagnosis.

## Conclusions

For ganglioneuromas, CECT and MRI are the main preoperative noninvasive diagnostic modalities, while the gold standard investigation remains histopathology. A lesson learned from this case is that preoperative ultrasonography or CT-guided biopsy is a necessity for definitive diagnosis and to delineate the line of management. Over-reliance on radiological findings for deciding the definitive line of management should be avoided. Cases like the present one should preferably be managed at a health center well-equipped with a catheterization lab and cavitron ultrasonic surgical aspirator (CUSA), by a team composed of surgical oncologists, vascular surgeons, and interventional radiologists. Subtotal or near-total excision of the tumor should be the goal of the surgery.
